# Clinical Toxicities of Histone Deacetylase Inhibitors

**DOI:** 10.3390/ph3092751

**Published:** 2010-08-26

**Authors:** Srividya Subramanian, Susan E. Bates, John J. Wright, Igor Espinoza-Delgado, Richard L. Piekarz

**Affiliations:** 1 TRI, Inc., 6500 Rock Spring Dr, Bethesda, MD 20817, USA; E-Mail: VSubramanian@tech-res.com (S.S.); 2 Center for Cancer Research, National Cancer Institute, Bethesda, MD 20892, USA; E-Mail: BatesS@mail.nih.gov (S.E.B.); 3 Cancer Therapy Evaluation Program, National Cancer Institute, Bethesda, MD 20892, USA; E-Mail: wrightj@ctep.nci.nih.gov (J.J.W.); EspinozaIg@mail.nih.gov (I.E.)

**Keywords:** histone deacetylase inhibitors, HDAC, toxicities, chemotherapy, clinical trial, HDI

## Abstract

The HDAC inhibitors are a new family of antineoplastic agents. Since the entry of these agents into our therapeutic armamentarium, there has been increasing interest in their use. Although this family comprises chemical compounds from unrelated chemical classes that have different HDAC isoform specificities, they surprisingly have very similar toxicity profiles. In contrast, the observed toxicity profile is somewhat different from that of traditional cytotoxic chemotherapeutic agents and from other epigenetic agents. While some of the side effects may be familiar to the oncologist, others are less commonly seen. As some patients remain on therapy for a prolonged period of time, the long-term sequelae need to be characterized. In addition, since preclinical models suggest promising activity when used in combination with other antineoplastic agents, combination trials are being pursued. It will thus be important to distinguish the relative toxicity attributed to these agents and be alert to the exacerbation of toxicities observed in single agent studies. Notably, few of the agents in this class have completed phase 2 testing. Consequently, more clinical experience is needed to determine the relative frequency of the observed side effects, and to identify and develop approaches to mitigate potential clinical sequelae.

## 1. Introduction

The histone deacetylase (HDAC) inhibitors (HDIs) are a new class of antitumor agents, generally considered to be members of a growing family of epigenetic modifying agents. These agents have been studied extensively in the laboratory due to their ability to reverse the neoplastic phenotype and exert tumoricidal activity. Early agents in this class were studied as differentiating agents but were not as potent or specific as the newer generation of inhibitors that exhibited promising activity in *in vitro* and xenograft models. Two of these, vorinostat, followed by romidepsin, have gained approval by the United States (U.S.) Food and Drug Administration (FDA) for the treatment of patients with refractory cutaneous T-cell lymphoma (CTCL).

Acetylation occurs as a post-translational modification on the ε-amino group of lysine residues of cellular proteins; histones being the most studied substrate. Described in more detail elsewhere, the histone acetyltransferases (HAT) are the group of enzymes responsible for acetylation of proteins and include p300, CREB-binding protein (CBP), PCAF, and TAF1 [1]. HDACs are the group of enzymes responsible for removal of the acetyl groups. They are generally divided into several classes including class I (HDAC1, HDAC2, HDAC3, and HDAC8), class II (HDAC4, HDAC5, HDAC6, HDAC7, HDAC9, and HDAC10), and class IV (HDAC11). Class III, commonly referred to as the sirtuins, comprise the NAD-dependent HDACs sirtuins (SIRT) SIRT 1–7. Agents that target the SIRT will not be discussed in this manuscript as none have yet entered clinical evaluation.

Several chemical classes of HDIs have been developed (Table 1). These include the short-chain fatty acids (SCFAs), such as sodium butyrate, phenylbutyrate, pivanex (pivaloyloxymethyl butyrate; AN-9), and valproic acid. Newer and more selective classes include hydroxamic acids such as vorinostat (SAHA), belinostat (PXD101), panobinostat (LBH589), and dacinostat (LAQ824); benzamides including entinostat (MS-275) and mocetinostat (MGCD-0103); and the bicyclic depsipeptide, romidepsin (FK228).

**Table 1:** Classification of HDAC inhibitors by chemical structure.

Short-chain Fatty Acids	Hydroxamic Acids	Depsipeptide	Benzamides
Sodium butyrate, Phenylbutyrate, Pivanex, Valproic acid	Vorinostat, Belinostat, Panobinostat, Dacinostat	Romidepsin	Entinostat, Mocetinostat

While all HDIs developed to date have activity against the class I HDAC enzymes, they have variable activity against the other HDAC isoenzymes. It is not known whether more selective HDIs would be more effective; that is, it is not known which of the HDAC isoenzymes are most important for the antineoplastic effects seen with these agents. In addition, it is not known whether more selective agents would have a better side effect profile. One of the key reasons for this is that the exact mechanisms of antitumor activity of these agents are not known. Across the numerous laboratory studies of HDIs, more than 10 possible mechanisms have been hypothesized [2].

As with any class of anticancer agents, the HDIs have associated toxicities, and physicians need to be aware of potential side effects. Some side effects can be routinely managed (e.g., anti-emetics to alleviate associated nausea and vomiting); some, however, require careful monitoring, including selection of patients prior to initiating therapy and monitoring laboratory parameters of patients undergoing treatment. There is still only limited clinical experience with HDIs, particularly in patients with co-morbidities and concomitant medications.

## 2. Safety Experience in Clinical Trials

HDIs have been well tolerated and appear to have a different toxicity profile compared to classical chemotherapeutic agents. The primary toxicities noted were nausea/vomiting, fatigue, and a transient decrease in platelet and white blood cell counts. Due to asymptomatic electrocardiogram (ECG) changes noted in early trials, patients receiving these agents have been closely monitored; however, HDIs do not appear to be associated with a greater incidence of cardiac adverse events than other chemotherapeutic agents. Vorinostat and romidepsin have been the most studied, followed by entinostat and belinostat. Table 2 lists the dose-limiting toxicities (DLTs) reported in phase I trials and Table 3 lists the adverse events reported in phase II trials. Data from 22 phase I single-agent trials and 12 phase II single-agent trials were reviewed and presented in these tables. Table 3 reports the rates of the more commonly reported adverse events observed in the 12 reports of Phase II studies reviewed here and thought to be at least possibly related to the HDI.

## 3. Common Adverse Events of HDAC Inhibitors from Single-agent Trials

### 3.1. Gastrointestinal

Nausea, vomiting, and anorexia were the most common higher grade adverse events observed with the use of HDIs (Table 3) [3,4,5,6,7]. Nausea and vomiting (up to 14% grade 3–4 reported in phase II trials) were managed using anti-emetic prophylaxis. Granisetron and lorazepam are effective in romidepsin-treated patients and are recommended [8,9]. Anorexia (up to 20% grade 3–4 in phase II trials) and dysgeusia can impact patients receiving long-term therapy [4,7,10]. Constipation, diarrhea, and dehydration were also seen in patients receiving HDIs [7,11,12,13]. For patients with dehydration, especially with nausea or anorexia, prophylactic administration of intravenous fluids appeared to help. Other, less common gastrointestinal events included grade 1–2 dry mouth [14], stomatitis [10], heartburn [15], and flatulence [15].

**Table 2:** Dose-limiting toxicities of HDAC inhibitors in phase I single-agent trials.

Agent	Dose-limiting Toxicities	Schedule	Ref.
Pivanex	None	6 h IV qd ×5; 21 d	[16]
Sodium Phenylbutyrate	Somnolence, confusion, hypokalemia, hyponatremia, hyperurecemia	120 h IV; 21 d	[17]
Sodium Phenylbutyrate	Short-term memory loss, sedation, confusion, nausea/vomiting	0.5–2 h IV BID d 1–5 & 8–12; 28 d	[18]
Valproic acid	Neurocognitive impairment, neuroconstipation, somnolence	1 h IV qd ×5; 21 d	[19]
Belinostat	Fatigue, elevated creatinine, elevated uric acid, decreased potassium, status epilepticus, paresthesia, vasculitis, renal failure	30 min IV qd ×5; 21 d	[20]
Belinostat	Fatigue, atrial fibrillation, nausea/vomiting, diarrhea	30 min IV qd ×5; 21 d	[21]
Dacinostat	Transaminase, fatigue, atrial fibrillation, elevated creatinine, QTc prolongation, febrile neutropenia, hyperbilirubinemia, death	3h IV d 1–3; 21 d	[22]
Panobinostat	QTcF prolongation	30 min IV d1–7; 21 d	[23]
Panobinostat	Diarrhea	Oral TIW; 28 d	[24]
Vorinostat	Dehydration, thrombocytopenia, diarrhea, fatigue, ALT/AST, anorexia, nausea/vomiting	Oral qd or BID or BID d 1–3 qw	[3]
Vorinostat	Fatigue, nausea/vomiting, diarrhea	Oral TID or BID ×14 d; 21 d	[25]
Vorinostat	Fatigue	Oral BID ×5 d qw or BID ×14 d q21d	[26]
Vorinostat	Thrombocytopenia, anorexia, fatigue	Oral BID ×14 d; 21 d	[27]
Romidepsin	Thrombocytopenia, fatigue	4 h IV d 1, 8 & 15; 28 d	[28]
Romidepsin	Fatigue, nausea/vomiting, thrombocytopenia, atrial fibrillation	4 h IV d 1 & 5; 21 d	[29]
Romidepsin	Hypocalcemia, sick sinus syndrome, asymptomatic T-wave inversion	4 h IV d 1, 8 & 15; 28 d	[30]
Entinostat	Nausea, vomiting, anorexia, fatigue	Oral q14d	[31]
Entinostat	Fatigue, LDH, hypertriglyceridemia, hyperglycemia, hypoalbuminemia, hypocalcemia, infection, anorexia, nausea, somnolence, weakness/unsteady gait	Oral qw ×2; 28 d or qw ×4; 42 d	[32]
Entinostat	Hypophosphatemia, hypoalbuminemia, hyponatremia	Oral qw ×4; 42 d	[33]
Entinostat	Asthenia, hypophosphatemia	Oral q14d or qw ×3; 28 d	[34]
Mocetinostat	Fatigue, nausea/vomiting, diarrhea, mucositis, acid reflux, gastritis, hip/leg pain	Oral TIW	[35]
Mocetinostat	Fatigue, nausea/vomiting, anorexia, dehydration	Oral TIW ×2; 21 d	[6]

ALT/AST: Alanine aminotransferase/Aspartate aminotransferase; BID: Twice daily; BIW: Twice weekly; d: Day; h: Hour; LDH: Lactate dehydrogenase; IV: Intravenous; min: Minutes; qd: Every day; q14d: Every 14 days; q21d: Every 21 days; qw: Every week; TIW: Three times a week

**Table 3:** Rate of common adverse events of HDAC inhibitors from phase II single-agent trials.

Agent (Disease)	Ref	n	Fatigue	Nausea	Vomiting	Anorexia	Weight Loss	Diarrhea	Creatinine	AST/ALT	Hypophosphatemia	Hyponatremia	Hypoalbuminemia	Hyperbilirubineia	Hyperglycemia	Hypocalcemia	Anemia	Thrombocytopena	Neutropenia	Asthenia
Pivanex (NSCLC)	[4]	47	34	17		9														13
Belinostat (mesothelioma)	[13]	13	15	15		15			8	8		23	8		46		46			
Vorinostat (CTCL)	[14]	37	73	49	24	22	27	49	16								11	54		
Vorinostat (CTCL)	[36]	74	46	43	12	26	20	49	15								12	22		
Vorinostat (DLBCL)	[37]	18	50	39	33	28	11	61				11			11		33	28		22
Vorinostat (breast, colorectal, or NSCLC)	[10]	16	62	62	56	81	50	56									19	50		31
Romidepsin (mNET)	[5]	15	74	86	67	73	27	33		34				27		34	46	59	7	
Romidepsin (RCC)	[38]	29	93	93	66	69		17	21			3			10	7	21	24	21	
Romidepsin (AML)	[39]	20	20		40	25				15										
Romidepsin (CTCL)	[40]	71	41	52	19	21		8		12	8	8	20	3	18	42	37	40	36	
Entinostat (melanoma)	[41]	28	11	21	11			11			29						4			
Vorinostat (thyroid carcinoma)	[15]	19	89	69		85	69	69	42	5	5	11	11	16	53	32	79	83	37	

Toxicities are in percentages, n: number of patients; AML: Acute myeloid leukemia; ALT/AST: Alanine aminotransferase/Aspartate aminotransferase; CTCL: Cutaneous T cell lymphoma; DLBCL: Diffuse large B cell lymphoma; mNET: Metastatic neuroendocrine tumors; NSCLC: Non-small cell lung cancer; RCC: Renal cell cancer.

### 3.2. Constitutional

Fatigue is a common side effect seen with all HDIs, and the symptom rapidly resolves upon drug discontinuation. Fatigue was one of the common DLTs seen in phase I trials [21,28,29]. In addition, up to 26% grade 3–4 fatigue was noted in phase II trials [15]. Of note, the time course of fatigue did not correlate with a change in hemoglobin levels. Grade 1–2 fever was another constitutional event observed in some phase I trials [21,29]. The symptoms of fever and fatigue could be a consequence of cytokine release. Samples from two patients showed an increase in interleukin (IL)-6 levels post-belinostat treatment [21]; however, the sample size was too small to allow meaningful conclusions. Weight loss (grade 1–2) has also been noted [14,36].

### 3.3. Hematologic

Thrombocytopenia, neutropenia, and anemia are observed following HDI administration; however, these effects are transient and reversible. Thrombocytopenia (grade 3–4: up to 50% in phase II trials) was the most common hematologic event observed, particularly with romidepsin and vorinostat [10,11,14,28,29,36,37,40,42,43]. Neutropenia (grade 3–4: up to 21%) was observed in some trials [11,12,43,44,45]. Febrile neutropenia was dose limiting in one phase I trial with dacinostat [22]. Anemia (grade 3–4: up to 21%) was also reported [10,13,28,33,38,40,45]. The neutropenia and thrombocytopenia resolve shortly after removal of the agent, with blood counts recovering to baseline within 10 days after treatment. Consistent with this lack of true myelosuppresion, bone marrow cells exposed *in vitro* were approximately one thousand-fold less sensitive to HDIs [46]. In a colony forming assay, less than 50% suppression of colony forming unit-granulocyte-macrophage (CFU-GM) was observed at 3 µM, the highest concentration of romidepsin tested. This compares favorably with the IC_50_ of 1.4 nM observed in a CTCL cell line and peak plasma concentration of 700 nM (with a significant fraction being protein bound) observed in CTCL patients [40,47]. Accordingly, the observed neutropenia and thrombocytopenia may be a cytokine-mediated activity rather than a direct toxic effect on the bone marrow, as noted above.

### 3.4. Cardiac

ECG changes, primarily characterized as T-wave flattening or inversions, were observed in clinical trials with HDIs [2,7,23,28,29,30]. To better characterize the incidence and potential clinical sequelae of these observed ECG findings, intensive cardiac monitoring was incorporated into the phase II trial of romidepsin for patients with T-cell lymphoma conducted at the National Institutes of Health (NIH) Clinical Center. Results of the cardiac monitoring studies performed have been reported elsewhere [9]. These studies focused on ECG changes, evaluating myocardial integrity, cardiac function, and evidence of potential dysrhythmia. Cardiac studies included serial ECGs, measurement of cardiac enzymes, echocardiograms, baseline 24-hour Holter analysis, and telemetry monitoring during the first dose of the first cycle. ECGs were obtained pre- and post-treatment to better determine the frequency and degree of changes; serum cardiac troponin I levels were obtained to look for evidence of myocardial damage; echocardiograms were obtained at the time of ECG changes to detect possible wall motion abnormalities; and serial ejection fraction (EF) evaluations were performed to evaluate potential long term changes in cardiac function. This testing revealed no evidence of acute or cumulative cardiac damage, based on serial troponin I values, Multi Gated Acquisition (MUGA) scans, or echocardiograms [9]. It is interesting to note that ECG changes are considered to be a drug-related event with other drugs as well. ECG changes consisting of T-wave flattening or inversions and ST-segment changes have been noted in patients taking phenylthiazines including thioridazine and chlorpromazine [48].

Arrhythmias have been reported in patients treated with HDIs; however, the relationship to the HDI is unclear. Patients observed to have these arrhythmias were noted to have risk factors for arrhythmias such as electrolyte disturbances. Additionally, this population of patients had undergone extensive prior therapy frequently including anthracyclines, and some patients had undergone stem-cell transplant. Atrial fibrillation has been the most common arrhythmia noted in patients treated with HDIs [5,13,38,40] and has been recorded as a DLT in phase I trials [21,22,29]. Other arrhythmias have also been occasionally noted. These have been described and reported for patients on trials with romidepsin, probably as a consequence of the intensive monitoring. In a pediatric romidepsin trial, one patient developed sick sinus syndrome, which was asymptomatic and reversible [30]. One study of romidepsin in patients with metastatic neuroendocrine tumors reported asymptomatic short episodes of ventricular tachycardia in two patients monitored after treatment with romidepsin; however, the significance is unclear as baseline monitoring was not performed [5]. Ventricular tachycardia was also noted in three of 71 patients with CTCL receiving romidepsin. Each patient was also found to have ventricular tachycardia or significant ventricular ectopy on pretreatment Holter monitor [40]. These patients were noted to have uncorrected deficiency of potassium or magnesium, either of which will predispose to arrhythmias. As a result, it has been suggested that special attention be paid to electrolyte levels, especially potassium and magnesium in patients receiving romidepsin and perhaps any HDI. Standard Cardiology guidelines recommend replacement of potassium and magnesium in patients at risk for arrhythmias [49]. Electrolyte replacement guidelines have been incorporated in romidepsin clinical trials.

As frequent ECGs were obtained, prolongation of QT interval was noted in some patients. The incidence of QT interval prolongation and whether these have clinical relevance is not known. It is thought that a small increase in the QT interval may be clinically significant if superimposed on some other factor such as baseline QT prolongation, administration of other agents that may also impact the QT interval or agents that may interfere with the metabolism of the drug [50]. In addition, the flattening of the T-wave and distortion of the ST-segment observed with the administration of HDIs may compromise the ability to properly evaluate the QT interval. Dose-limiting grade 3 asymptomatic QTcF prolongation was noted in four patients in a panobinostat trial [23]. The QTcF effect was reversed upon treatment termination. In addition, QT interval prolongation was also noted in patients treated with dacinostat [51] and vorinostat [10,36,37]. QT interval prolongation was also noted in the trial of romidepsin for pediatric patients [30]. In the trial of patients with neuroendocrine tumors, three patients were noted to have QT interval prolongation [5]. Again, in the phase II trial of romidepsin with extensive cardiac monitoring, QT interval prolongation was noted to occur in some patients after treatment, with a median increase of 14 msec [9]. In this analysis, where romidepsin was administered weekly, 3 out of 4 weeks, QTc prolongation was noted to reverse within 48 hours of administration. After independent central review, this effect on the QT interval appears to be nearer a mean of 5 msec (from baseline pre-anti-emetic to post-romidepsin treatment); this is the effect on the QT interval reported with the administration of ondansetron alone [52,53]. QTc effects may be of greater concern for agents administered on a more frequent schedule or on a continuous basis. More significantly, genetic factors may play a significant role in predisposition to drug effects, as has been previously noted [54,55,56]. In patients treated with romidepsin, approximately 19% of the patients had no evidence of QT interval prolongation.

In summary, the ECG changes observed to date with HDI treatment appear to be clinically insignificant; however additional studies may be needed to rule out any long-term cardiac effects.

### 3.5. Metabolic

Liver toxicities, including elevations in liver transaminases (grade 3–4: up to 7% in phase II trials) and grade 1–2 hyperbilirubinemia and hypoalbuminemia were reported [5,13,22,33,39,40]. Electrolyte imbalances such as hypocalcemia (grade 3–4: up to 11%) or hyponatremia (grade 3–4: up to 23%) have been reported, as has mild hypokalemia or hypophosphatemia [6,13,15,17,20,30,33,34,36,37,40,42]. Renal dysfunction in the form of grade 1–2 elevated creatinine levels and hyperuricemia have been infrequently reported with HDIs [17,20,36,42]. Elevated lactate dehydrogenase (LDH), hypertriglyceridemia, and hyperglycemia were reported as DLTs following treatment with entinostat [32]. Tumor lysis accompanied by a metabolic profile (hyperkalemia, hyperphosphatemia, hyperuricemia, and renal insufficiency) and a modest, albeit transient, decrease in tumor burden has been reported in some patients treated with HDIs [8,20].

### 3.6. Other Side-Effects

Neurological events were noted in earlier studies with the short chain fatty acids such as sodium butyrate, more recently in studies with valproic acid, another short chain fatty acid [19], and in trials using newer agents. The events included status epilepticus in association with uremia and paresthesia with belinostat [20]; dose-limiting somnolence and unsteady gait with entinostat [32], and somnolence and confusion with phenylbutyrate [17]; and confusion, neuroconstipation, and somnolence with valproic acid [19]. These effects occurred at doses higher than the maximum tolerated dose (MTD). Grade 3 ataxia (5%) and grade 1–2 neurological events including vertigo and memory loss were observed in phase II trials with vorinostat [10,15].

Mild (grade 1–2) cough and dyspnea were noted in some trials [4,13,15]. In one phase I trial, 25 patients (34%) experienced dyspnea without associated cardiopulmonary or imaging abnormalities [3]. Grade 3 hypoxia was observed in two patients on a romidepsin trial; however, the investigators attributed this event to disease progression [45]. Grade 2–3 bronchitis/pneumonia was observed in three patients (11%) on a vorinostat trial [15].

Infections occurred in 38 CTCL patients (54%) on a romidepsin trial [40]; these included bacterial infections of the skin, upper and lower respiratory tracts, gastrointestinal and urinary tracts, and bacteremia. Two patients died of sepsis within 30 days of removal from the study as a result of disease progression. Of note, patients with CTCL have a high incidence of infections as a feature of their illness [57]. Great care should be observed in the use of indwelling venous access in patients with CTCL. Skin preparation with topical antiseptic, prophylactic antimicrobial treatment, and immediate removal of indwelling central venous access after administration of each dose is strongly recommended for patients with extensive skin involvement of their CTCL. Grade 2 infection was also noted in a small belinostat trial [13]. Lung infection/pneumonia were rarely observed [7,15].

Grade 3–4 venous thromboembolic events and pulmonary embolism (grade 3–4: up to 10%) were noted in some phase II trials [14,15,36,38,45]. Relatively uncommon adverse events included pain [10,41], muscle spasms [36], alopecia [36,37], and nail changes [20].

### 3.7. Death

Deaths have been reported in studies with HDIs. Table 4 lists the on-study deaths reported in more than 30 phase I and phase II studies reviewed here, which included over 1,000 patients. It is not clear that the incidence observed is any higher than that usually observed in trials of experimental therapeutics for cancer [58,59,60]. Upon review of on-study deaths with romidepsin, it was noted that these patients had risk factors for sudden death prior to enrollment. Key factors (Table 5) in minimizing potential risks are the exclusion of patients at risk for sudden death, monitoring electrolyte levels, and avoiding the use of agents that prolong the QT interval or may interfere with drug metabolism [61].

**Table 4:** Deaths reported on trials with HDIs. (**A**). Phase I trials; (**B**). Phase 2 trials.

**(A).** Phase I trials.			
**Agent**	**Ref.**	**Cause of Death Reported**	**Reported as Possibly Drug-Related**
Belinostat	[20]	Disease progression	No
Dacinostat	[22]	Atrial fibrillation and acute renal failure	Yes
Panobinostat	[23]	Sepsis	No
Vorinostat	[62]	Acute cardiac event (patient with cardiopulmonary disease)	No
Vorinostat	[3]	Infection	No
Vorinostat	[25]	Unknown	No
Entinostat	[32]	Disease progression (n = 2)	No
		Progressive fungal pneumonia (n = 3)	No
		Sepsis (n = 3)	No
		Sudden death (patient with heart disease developed diarrhea and dehydration due to *C. difficile* colitis and pancreatic insufficiency)	No
**(B).** Phase 2 trials.			
**Agent**	**Ref.**	**Cause of Death Reported**	**Reported as Possibly Drug-Related**
Belinostat	[13]	Withdrawal of supportive care	Yes
Vorinostat	[14]	Disease progression	No
		Sepsis	No
Vorinostat	[36]	Disease progression	No
		Ischemic stroke	No
		Unexplained (patient with hypertension and valvular heart disease)	No
Vorinostat	[37]	Disease progression	No
		Acute myocardial infarction	No
Vorinostat	[10]	Disease progression (n = 2)	No
		Tumor hemorrhage	No
		General health deterioration	No
		Cachexia	No
Romidepsin	[5]	Sudden death (patient with cardiomegaly with biventricular hypertrophy)	Yes
Romidepsin	[38]	Sudden death	Yes
Romidepsin	[43]	Disease progression	No
Romidepsin	[40]	Sudden death (patient with hypertrophic cardiac disease with significant valvular pathology)	Yes
		Sepsis (n = 2)	Yes, No
Entinostat	[41]	Disease progression	No

**Table 5:** Cardiac exclusion criteria and on-study monitoring.

**A. Prototypic cardiac exclusion criteria**
Uncontrolled hypertension
Active coronary artery disease
Myocardial infarction or unstable angina within the past 6 months
Prolonged QTc on screening ECG
Congenital long QT syndrome
Wolff-Parkinson-White syndrome
History or presence of sustained ventricular tachycardia
History of ventricular fibrillation or Torsades de Pointes
Heart block
Cardiomyopathy: Dilated, hypertrophic, or restrictive
New York Heart Association class III-IV congestive heart failure
**B. Cardiac monitoring during therapy**
Avoidance of agents known to prolong the QTc
Avoidance of agents that may interfere with metabolism
Monitor and replace electrolytes to maintain serum potassium ≥4.0 mmol/L and serum magnesium ≥0.85 mmol/L prior to administration of drug.

ECG: Electrocardiogram
